# Incidental visual processing of spatiotemporal cues in communicative interactions: An fMRI investigation

**DOI:** 10.1162/imag_a_00048

**Published:** 2023-12-18

**Authors:** Anthony P. Atkinson, Quoc C. Vuong

**Affiliations:** Department of Psychology, Durham University, Durham, United Kingdom; Biosciences Institute and School of Psychology, Newcastle University, Newcastle upon Tyne, United Kingdom

**Keywords:** social interaction, biological motion, person perception, neuroimaging, superior temporal sulcus, parietal cortex

## Abstract

The interpretation of social interactions between people is important in many daily situations. The coordination of the relative body movements between them may provide visual cues that observers use without attention to discriminate such social interactions from the actions of people acting independently of each other. Previous studies highlighted brain regions involved in the visual processing of interacting versus independently acting people, including posterior superior temporal sulcus, and areas of lateral occipitotemporal and parietal cortices. Unlike these previous studies, we focused on the *incidental* visual processing of social interactions; that is, the processing of the body movements outside the observers’ focus of attention. In the current study, we used functional imaging to measure brain activation while participants were presented with point-light dyads portraying communicative interactions or individual actions. However, their task was to discriminate the brightness of two crosses also on the screen. To investigate brain regions that may process the spatial and temporal relationships between the point-light displays, we either reversed the facing direction of one agent or spatially scrambled the local motion of the points. Incidental processing of communicative interactions elicited activation in right anterior STS only when the two agents were facing each other. Controlling for differences in local motion by subtracting brain activation to scrambled versions of the point-light displays revealed significant activation in parietal cortex for communicative interactions, as well as left amygdala and brain stem/cerebellum. Our results complement previous studies and suggest that additional brain regions may be recruited to incidentally process the spatial and temporal contingencies that distinguish people acting together from people acting individually.

## 1 Introduction

Making sense of other people’s actions towards each other is a pervasive and fundamental aspect of being human. For example, people may jointly act towards a common goal (e.g., lifting a heavy object) or engage in communicative actions with each other (e.g., gesturing for help to lift a heavy object). Clearly, social interactions between people are qualitatively different from the independent actions of individual people who happen to be in spatial proximity to each other (e.g., a person lifting a box, and another person close by drinking from a water bottle). A growing body of research demonstrates that we readily make sense of social and communicative encounters, and form first and lasting impressions of the interacting agents and of the groups they comprise (Papeo, 2020; Quadflieg & Koldewyn, 2017; Quadflieg & Penton-Voak, 2017). Indeed, there is an emerging consensus based on behavioural and neuroimaging evidence that the human visual system processes interacting agents more as a unified whole or Gestalt, rather than processes each agent independently and then combines their actions to infer the interaction (e.g., Abassi & Papeo, 2020; Ding et al., 2017; Landsiedel et al., 2022; Papeo, 2020; Vestner et al., 2019; Walbrin & Koldewyn, 2019; Yin et al., 2018). Further evidence that interacting agents are special comes from functional neuroimaging studies showing that human as well as monkey brains contain a network involved in and perhaps even specialised for the visual processing of interacting others (Centelles et al., 2011; Georgescu et al., 2014; Isik et al., 2017; Landsiedel et al., 2022; Sapey-Triomphe et al., 2017; Sliwa & Freiwald, 2017; Walbrin et al., 2018).

In contrast to previous fMRI studies investigating the brain regions subserving visual processing of social interactions, our focus is on the *incidental* visual processing of social interactions. By incidental processing, we mean the visual processing of the stimuli of interest outside the participant’s task-related focus of attention. In a face perception study, for example, participants might be required to classify the sex of faces that also vary in their emotional expression, and yet behavioural and neuroimaging evidence can be acquired to show that information about the emotional expression has nevertheless been extracted from the faces (e.g., Atkinson et al., 2005; Gorno-Tempini et al., 2001; Phillips et al., 1998). In the present study, the stimulus property of interest was whether human dyads were interacting or not, yet the task required participants to focus attention on and respond to different stimuli on the screen, namely, the relative brightness of two fixation crosses.

Some studies investigating the visual processing of interacting dyads have required participants to judge or otherwise think about the social or interactive nature of the stimuli (Centelles et al., 2011; Georgescu et al., 2014; Kujala et al., 2012; Sapey-Triomphe et al., 2017). Other studies have required participants to judge the viewed movements without instruction to attend specifically to the social or interactive nature of those movements (Petrini et al., 2014: judging whether the current display is the same as or different from the previous one), or simply to passively view the stimuli (Isik et al., 2017; Landsiedel et al., 2022; Walbrin & Koldewyn, 2019; Walbrin et al., 2018, 2020, 2023). Passive viewing of the stimuli, as well as tasks requiring participants to make a judgement about the viewed stimuli that is not orthogonal to their interactive nature, provide ample opportunity for the participants to think about the nature of the bodies or bodily movements, including whether and how the depicted people are interacting, even if they are not directly instructed to do so.

A few studies have required participants to engage in a task unrelated to the nature of the viewed bodies or bodily movements, but participants were not engaged on the orthogonal task on every stimulus presentation or trial (e.g., Abassi & Papeo, 2020, 2022; Bellot et al., 2021). Across the entire experimental run, participants may have time to attend to and think about the nature of the bodies or bodily movements, even if they are not directly instructed to do so. These studies also had participants fixate the centre of the screen. Although this manipulation helps to minimise eye movements, it may affect how participants process social and non-social interactions. For example, in Abassi and Papeo’s (2020, 2022) fMRI experiments, participants were instructed to attend to a central fixation cross throughout the experiment and to detect and respond to its change in colour that occurred on 37% of the stimulation or fixation periods; the stimuli of interest were static body images, one located either side of the fixation cross. In Bellot et al.’s (2021) fMRI experiment, participants were instructed to fixate the centre of the screen and to detect the occasional colour change of the dots in the nearby point-light^1^ dyads—a change that occurred on only 2.5% of trials.

What brain regions, if any, are involved in the incidental processing of social interactions and what visual information might they be operating over? In this study, we address these questions. To do so, we used a same-different discrimination task orthogonal to the social nature of the stimuli on every trial. This explicit judgement task does not require participants to make a judgement about or simply attend to the interactive nature of the stimuli, and restricts the opportunity for participants to think about the interactive nature of those stimuli, particularly during the early period when the communicative gesture is performed. More specifically, the participants’ task was to judge whether two fixation crosses were the same or different shades of gray. This required participants to direct their eyes between the locations of two point-light agents who were interacting or acting independently but to attend to and make a judgement about different stimuli also at the locations of the two agents. The point-light displays and the movements, actions, or interactions they depicted were thus task-irrelevant.

What visual information might such incidental processing of social interactions be operating over? Many studies demonstrate that observers quickly and accurately distinguish interacting from non-interacting dyads and interpret those interactions based solely on visual motion cues, even with facial and static body-form cues and information about the wider scene context removed; for example, by using point-light agents (e.g., Centelles et al., 2013; Manera et al., 2010, 2011, 2013; 2015; Neri et al., 2006; Piwek et al., 2016; Thurman & Lu, 2014; von der Lühe et al., 2016). Behavioural evidence has shown that the visual cues that observers use to detect and distinguish between different types of social interactions include the local motion (especially velocity) of body parts, notably of the arms, feet, and hips (de la Rosa et al., 2013), and total motion energy (Thurman & Lu, 2014), but also particularly spatiotemporal contingencies between the actions of the agents (Manera et al., 2013; Neri et al., 2006; Thurman & Lu, 2014). In the current study, we implemented 2 stimulus manipulations to vary the availability of different types of visual cues in point-light displays of interacting dyads. The first stimulus manipulation involved changing the facing direction of one of the agents in the dyad. In the unmanipulated point-light display, the interacting agents are facing each other, whereas in the “nonfacing” versions of the same displays, one of the point-light agents was rotated such that it faced away from, rather than towards, the other agent. This facing manipulation preserves temporal features, including the local motion trajectories of each agent and any incidental temporal contingencies between the two agents (e.g., Agent A’s elbow happened to move when Agent B’s knee moved). Critically, however, this facing manipulation disrupts spatiotemporal contingencies between the two agents for communicative interactions because of the spatial change in facing direction. For example, if Person A gestures to Person B to approach, the gesture is only communicative if Person A faces Person B. Brain regions sensitive to these spatiotemporal contingencies are hypothesised to show activation to interacting compared to independently acting dyads when the interacting individuals are facing each other but not when they are not facing each other.

The second manipulation involved generating scrambled versions of the point-light stimuli, in which the starting positions of each point-light dot were randomly shifted along the vertical axis. This scrambling is typically used with point-light displays to disrupt the processing of configural information about the spatiotemporal relationships between the dots (within an agent), and thus the perception of coherent motion, people, and their actions in the point-light displays, whilst preserving local dot motion (e.g., Cutting et al., 1988; Peuskens et al., 2005; Troje, 2013). It is often used to functionally localise biological-motion regions, particularly posterior STS (e.g., Grossman et al., 2000; Saygin et al., 2004). In our study, this manipulation further allowed us to set up a contrast in the fMRI analysis in which we compared the activation elicited by communicative interactions versus independent actions after having subtracted out the contributions of their respective local dot motions. Such a contrast was expected to show brain regions involved in extracting information related to motion-mediated structure, including spatiotemporal contingencies between body parts within a single body, as well as spatiotemporal contingencies between the parts of different bodies.

What brain regions might be engaged by incidental processing of visual cues for social interactions? Posterior superior temporal sulcus (STS), particularly in the right hemisphere, is a candidate region, given its central role in the attended visual processing of social interactions (Bellot et al., 2021; Centelles et al., 2011; Georgescu et al., 2014; Isik et al., 2017; Landsiedel et al., 2022; Sapey-Triomphe et al., 2017; Walbrin & Koldewyn, 2019; Walbrin et al., 2018, 2020). Yet the response of posterior STS to single-agent biological motion is strongly suppressed when it is not task-relevant (Jastorff & Orban, 2009; Safford et al., 2010). This latter finding casts doubt on the hypothesis that incidental processing of interacting others will recruit posterior STS. Other candidate regions include the body-selective extrastriate body area (EBA) and the visual motion processing area V5/MT that overlaps EBA, given neuroimaging evidence that these regions also support the attended visual processing of social interactions (Abassi & Papeo, 2020, 2022; Gandolfo et al., 2023; Georgescu et al., 2014; Landsiedel et al., 2022; Walbrin & Koldewyn, 2019). Further candidate regions for incidental processing of visual cues for social interactions are early visual cortices, which have been implicated in the processing of biological motion, especially in the extraction of motion or spatiotemporal cues more than of static form cues (e.g., Chang et al., 2018; Cracco, Lee, et al., 2022; Cracco, Oomen, et al., 2022; Duarte et al., 2022; Servos et al., 2002; Vaina et al., 2001). Indeed, areas V2 and V3 are involved in the extraction of structure-from-motion more generally (Murray et al., 2003; Orban et al., 1999; Paradis et al., 2000; Vanduffel et al., 2002). Additionally, the precuneus has been implicated in the attended perception of biological motion depicted in point-light displays of single people (e.g., Vaina et al., 2001), in the observation of two people interacting in realistic movie clips (Iacoboni et al., 2004), and in the attended (e.g., Sapey-Triomphe et al., 2017) and, most notably, in the unattended (Petrini et al., 2014) perception of socially interacting dyads depicted in point-light displays. Finally, more lateral regions of parietal cortex, particularly in intraparietal sulcus and surrounding regions of inferior and superior parietal lobules, have also been implicated in the extraction of 3D structure-from-motion (Murray et al., 2003; Orban et al., 1999; Vanduffel et al., 2002), in the attended perception of biological motion depicted in point-light displays of single people (e.g., Grèzes et al., 2001), and in the attended perception of socially interacting dyads depicted in point-light displays (e.g., Sapey-Triomphe et al., 2017).

In summary, in the current study, we functionally scanned observers whilst they were presented with point-light displays of agents communicating with each other or acting independently of each other but in the same spatial proximity. To investigate incidental visual processing of communicative compared to independent actions, we had the participants engage in a task requiring visual attention to the location of the point-light agents but that was unrelated to the point-light stimuli themselves. Importantly, (1) by including conditions in which the facing direction of one of the agents was reversed, and (2) by including scrambled versions of each type of action, we could test for brain regions sensitive to differences in visual cues related to global motion, including structure-from-motion information, between the communicative interactions and independent actions, and we could test for brain regions sensitive to the spatiotemporal contingencies of the two agents.

## 2 Methods

### 2.1 Participants

Thirty-three people (20 females, 13 males; mean age = 28.7 years, SD = 8.2, range = 20-51 years) took part in this experiment, comprising students and staff from Durham and Newcastle universities, including the two authors. Apart from the authors, the participants were compensated with vouchers from an online retailer to the value of £20 and were reimbursed travel costs. All participants had normal or corrected-to-normal vision. Vision correction was achieved either via contact lenses or MRI-compatible prescription glasses. All participants provided signed, informed consent, and were debriefed at the end of the study. The study was approved by the Durham University’s Department of Psychology Ethics Subcommittee.

### 2.2 Stimuli

The visual stimuli comprised point-light displays selected from the Communicative Interaction Database (Manera et al., 2010, 2015). Each selected display showed two point-light people facing each other, in profile-view. There were 7 communicative interactions (labelled in the database as: “choose which one”, “come closer”, “look at the ceiling”, “pick this up”, “sit down”, “squat down”, and “no”), in which the agent on the right gestured to the agent on the left who performed an action consistent with the gestured instruction. There were also 7 individual (i.e., non-communicative) actions, in which the 2 people performed independent actions (labelled as: “drink” (other agent sits down), “jump” (other agent picks something up), “lateral step” (other agent takes something and eats it), “look under the foot” (other agent moves something), “sneeze” (other agent turns around), “stretch” (other agent moves something), and “turn over” (other agent squats down)). We selected these stimuli from the larger set in the Communicative Interaction Database, based on the data from 140 participants across 7 cultures/languages published in Manera et al. (2015), such that they were all highly recognisable as either communicative interactions or individual actions and that classification accuracy did not differ between these 2 stimulus types: mean for communicative interactions = 90.4% (SD = 8.9%, range: 73.6-98.6%), mean for individual actions = 87.0% (SD = 7.9%, range: 72.1-96.4%), *t*(12) = 0.75, *p* = .47. The mean durations of the selected point-light displays were 4.15 s (SD = 0.54) for the communicative actions and 4.52 s (SD = 0.56) for the individual actions, *t*(12) = 1.29, *p* = .22.

For each stimulus, we generated a “nonfacing” version, in which the agent on the right was rotated 180^o^ along the vertical *z*-axis so that they faced away from, rather than towards, the agent on the left. For the communicative interactions, the righthand person performed the communicative gesture. This rotation resulted in a small shift of the point-light displays along the horizontal *x*-axis. Finally, we generated scrambled versions of all the stimuli from the communicative and individual action conditions (i.e., the facing versions), in which the starting position of each point-light dot was randomly shifted along the vertical axis. Thus, there were 6 conditions: 2 stimulus types (communicative, individual) x 3 stimulus conditions (facing, nonfacing, scrambled).

Lastly, we tested whether there were low-level motion differences between the selected sets of communicative interactions and individual actions. We therefore calculated total motion energy for each communicative and individual action. This involved computing, for each action, the optic flow vector at each local dot of the two agents for each consecutive frame using the Lucas and Kanade (1981) method as implemented in Matlab (version 2020a, with the Computer Vision Toolbox version 9.2). The position of the local black dots on each video frame (image) was derived from the *x*- and *y*-coordinate of the centroid of each dot. Because there is some variability in the optic flow from trial to trial for the same stimulus (e.g., due to the starting frame location or scrambling manipulation), we pre-generated 10 videos (variants) for each of the 2 stimulus types x 3 stimulus conditions point-light dyads (total of 60 pre-generated videos). We calculated both the maximum magnitude and mean magnitude of the vectors across all local dots and frames. The maximum and mean values were then averaged across actions and compared between the 2 stimulus types. For the facing stimulus condition, there were no significant differences in low-level motion between the 2 stimulus types. The maximum magnitude was *M* = 2.90 pixels, *SE* = .29 pixels for the communicative actions, and *M* = 2.76 pixels, *SE* = .22 pixels for the individual actions, *t*(12) < 1.0. Similarly, the mean magnitude was *M* = .36 pixels, *SE* = .05 pixels for communicative actions and *M* = .36 pixels, and *SE* = .03 pixels for individual actions, *t*(12) < 1.0.

**Fig. 1.**
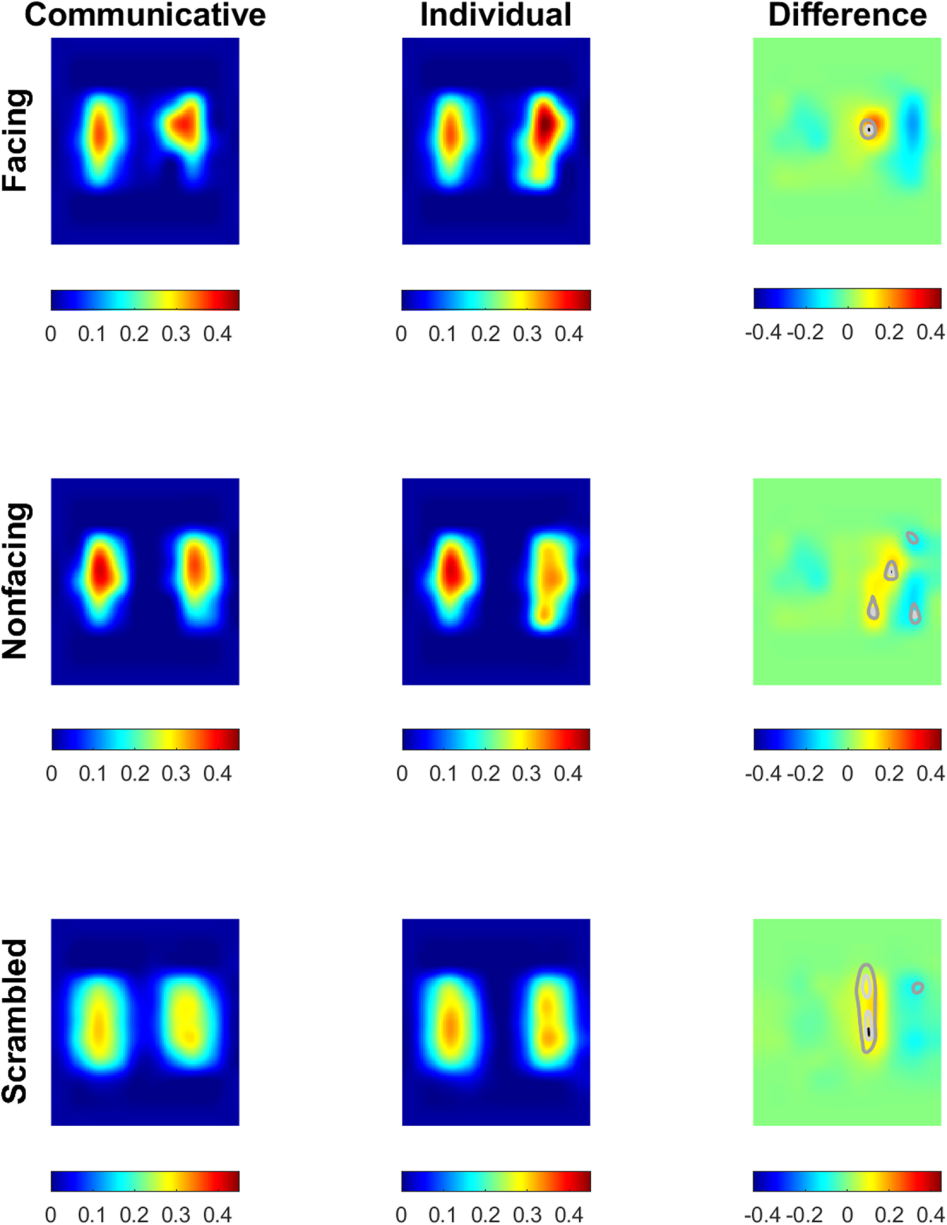
The regional motion energy for the communicative, individual, and scrambled point-light actions.

The maximum and mean magnitude of optic-flow vectors may not reflect any spatially local regions. We therefore divided the images into grids before calculating the regional mean magnitude of the optic-flow vectors. Each frame was 660 x 600 pixels, so we created an 11 x 10 grid (60 x 60 pixels per grid). Figure 1 shows the regional mean motion energy for the different actions, after scaling the resulting images using *bicubic* interpolation. We then calculated the difference between the communicative and individual actions for facing, nonfacing, and scrambled conditions, and tested for any regional differences in mean motion energy using *t*-tests. The contours in the difference images show some regional differences for the right actor (white - *p* < .001, mid-gray - *p* < .01, and dark-gray - *p* < .05, uncorrected).

**Fig. 2.**
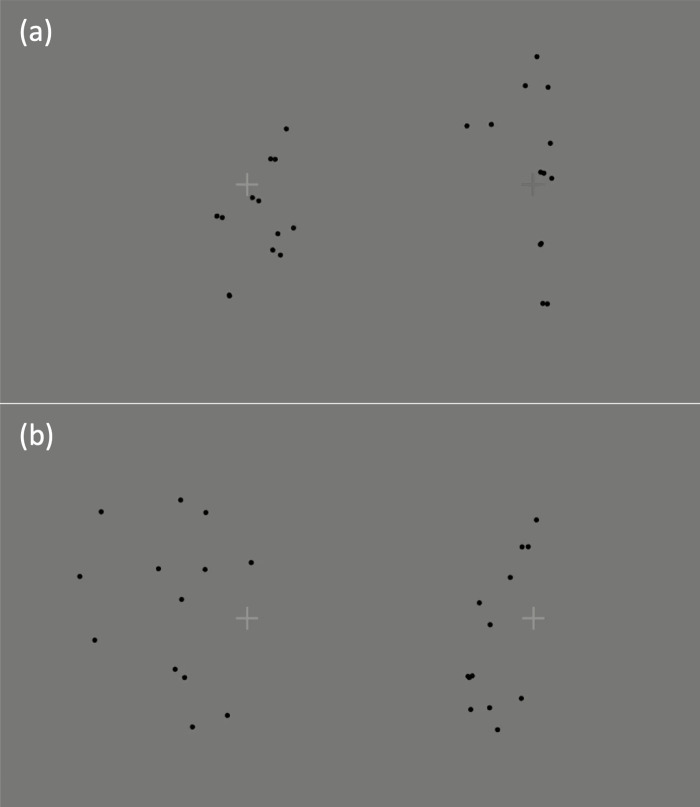
Still images from example point-light stimulus displays (images cropped for display purposes). The participant’s task was to decide whether the fixation crosses were the same or different colours/shades of gray. The point-light displays—which showed communicative interactions (as shown in panel a) or non-communicative (individual) actions, or their spatially scrambled counterparts (as in panel b), or counterparts in which the agent on the right was facing away from, rather than towards, the agent on the left—were thus task-irrelevant. Task difficulty was varied across trials by varying the relative gray-levels of the fixation crosses.

All point-light stimuli were displayed as black dots against a plain gray background (see Fig. 2). In addition to the point-light dyads, two fixation crosses were presented 200 pixels (2.2^o^) to the left and right of the centre of the display (i.e., 4.4^o^ apart), such that each was located approximately at the middle of one of the point-light figures on the first frame (see Fig. 2). The fixation crosses measured 64 pixels (0.7^o^) in width and height, and each point-light figure subtended ~4.1^o^ of visual angle vertically from head to foot dots (when standing at full height). The background screen colour was set to dark gray (intensity = 100). On “same” trials, the brightness of both fixation crosses was set to mid-gray (intensity = 128). On “different” trials, the brightness of one of the fixation crosses was set to mid-gray. The brightness of the other fixation cross was intensity = 128 +/- delta, where delta = 20 + floor(RAND*70) using MATLAB’s uniform random number generator. Thus, the minimum difference in brightness between the two fixation crosses was +/-20 and the maximum was +/-90. The sign of delta and the location of the mid-gray fixation (left or right) were also randomly determined on each trial.

### 2.3 Apparatus

The Biomotion toolbox (van Boxtel & Lu, 2013) was used to generate all point-light displays in real time on each trial. The point-light displays were presented from an orthographic perspective with the virtual camera placed to present a profile (side) view of the two agents (see Fig. 2). The stimuli were displayed on an MRI-compatible 24-inch LCD display (BOLDscreen; Cambridge Research Systems, UK) that was viewed through a mirror mounted on the MRI scanner’s head coil. This colour monitor has a resolution of 1920 × 1200 pixels, a viewable screen size of 518 × 324 mm, a 60 Hz frame rate, and a typical contrast ratio of 1000:1. The mirror-to-eyes distance is ~11 cm and the mirror-to-monitor distance 128 cm, giving a total viewing distance of 139 cm. Stimulus presentation and response collection were controlled with custom code in Matlab (MathWorks, Natick, MA) with the Psychtoolbox extension (Brainard, 1997; Kleiner et al., 2007; Pelli, 1997) and the Biomotion toolbox (van Boxtel & Lu, 2013).

### 2.4 Design and task

The presentation of the point-light stimuli was blocked according to stimulus type and condition, resulting in 6 task blocks: communicative facing, individual facing, communicative non-facing, individual non-facing, communicative scrambled, and individual scrambled. Each block contained one presentation of each of the 7 stimuli in that condition, presented in a new random order for each participant. To ensure that the duration of all point-light displays was similar, the maximum duration was set at 5.0 s (this clipped 2 individual action videos, and 1 communicative interaction with durations ~= 8.5 s). The last few seconds (~3.5 s) of the 3 clipped videos included the agent on the left of the screen returning to a “neutral” position, similar to how that agent started in the video (e.g., return to a neutral standing position). For the clipped communicative interaction video, some of the left agent’s action in response to the right agent’s gesture extended beyond 5 s and so was lost. Thus, each point-light display had a duration between 3.6 s and 5.0 s, and each presentation (trial) was separated by an interval (a blank gray screen) of either 0.7 s (for the communicative actions) or 0.5 s (for the individual actions). The interstimulus intervals for the two types of action were set at these different values to equate the duration of the task blocks, given that the total durations of the 7 communicative and 7 individual actions were 29.02 s and 31.63 s respectively. Thus, each task block, including interstimulus intervals, had a duration of 36 s.

Each imaging run comprised 10 task blocks, consisting of 2 of each of the 4 intact-stimuli blocks plus 1 block each of the two types of scrambled stimuli. Task blocks were presented in a new random order for each participant, and were interspersed with 14 s-long rest periods, indicated by the word “rest” in white in the centre of the monitor screen, which was otherwise blank (the same gray background as for the point-light displays). Each run of the experiment began and ended with the same 14 s-long rest block. Thus, the duration of each imaging run was 514 s. All participants bar two received 4 imaging runs; the other two participants received 3 runs (due to time constraints).

On each trial, participants were required to decide whether the two fixation crosses are the same or different colour (shade of gray) and to indicate their response by pressing the instructed button on a 5-key button box. They were instructed to respond as accurately as possible to the fixation task, and that the video may continue to play after they made their response. The response buttons were those placed under the index and middle fingers of their right hand. Button-response mappings were counterbalanced across participants.

### 2.5 MRI data acquisition

The study was conducted at Durham Centre for Imaging’s MRI facility at the James Cook University Hospital, Middlesbrough, England. Structural and functional MR images were acquired on a 3-Tesla Siemens Tim Trio scanner (Erlangen, Germany), fitted with a 32-channel head coil. Whole-brain T2*-weighted echo-planar images (EPI) with BOLD contrast were acquired. Each functional volume contained 32 axial slices, with 3 mm thickness, 0.99 mm gap, and in-plane resolution of 3 × 3 mm, acquired parallel to the length of the temporal lobes in a continuous sequence, with repetition time (TR) = 2000 ms, echo time (TE) = 35 ms, flip angle = 90°, field of view (FOV) = 192 × 192 mm, and acquired matrix of 64 × 64 voxels, reconstructed with matrix 64 × 64, and GRAPPA accelerator factor of 2. For each participant, 260 functional volumes (520 s) were collected for each of either 3 runs (2 participants) or 4 runs (31 participants) of the main experiment. An additional 4 “dummy” volumes were acquired at the beginning of each functional run to allow for signal equilibration, and were automatically excluded from the saved data files. For 26 of the 33 participants, B0 fieldmaps with the same dimensions as the functional images were also acquired to correct for static magnetic field inhomogeneities in the EPI images (GRE, 2D, TR = 468 ms, short TE = 4.92 ms, long TE = 7.38 ms, flip angle = 60°); however, these were not subsequently used in the preprocessing (see below). Prior to the functional and any field-mapping scans, anatomical T1-weighted images were acquired, with TR = 2.25 ms, TE = 2.52 ms, in-plane resolution of 0.5 × 0.5 mm, slice thickness = 1 mm, 192 slices, flip angle = 9°, FOV = 128 × 128 mm, acquired matrix of 256 × 256 voxels, reconstructed with matrix 512 × 512, and GRAPPA accelerator factor of 2.

### 2.6 MRI data quality checking and data exclusion

Prior to preprocessing the data, we generated visual quality reports for the anatomical and functional images with MRIQC (Esteban et al., 2017). MRIQC generates both individual reports for each scan of each participant and group reports, from which outliers can be identified. Following inspection of these reports, the following data exclusions were implemented. For one participant, 2 of their 4 imaging runs were excluded due to excessive head movement (defined as >10% of volumes in a run with a framewise displacement value >0.9; see below for further details of this measure). Two other participants had a small number of quality metric values, related to head motion, for 2 or 3 of their runs that were high relative to those from other runs and participants; we retained the data for these runs, however, because they were not considered to be substantial outliers and additional measures were subsequently implemented during data processing to compensate for such head motion artifacts (see below). Additionally, 1 run for another participant was excluded from analyses because the data acquisition was out of sync with the stimulus presentations. And for another participant, only the first 227 of the scheduled 260 volumes were able to be collected from their first run; that is, this run ended after 4 s of the final inter-block rest period, and thus data for the remaining 10 s of that rest period, and for the final task block and final rest period, were missing. We nevertheless included the data for this run in the analyses. In summary, the analyses were performed on data from 33 participants with a total of 127 imaging runs: 29 participants × 4 runs (1 of whom had 1 run with a missing task block), 3 participants × 3 runs, and 1 participant × 2 runs.

### 2.7 MRI data preprocessing

Results included in this manuscript come from preprocessing performed using fMRIPrep 22.1.1 (Esteban et al., 2019; RRID:SCR_016216), which is based on Nipype 1.8.5 (Gorgolewski et al., 2011; RRID:SCR_002502). This preprocessing was run on Durham University’s high-performance computing cluster. We here summarise the preprocessing steps performed by fMRIPrep. The full description of this preprocessing (comprising the boilerplate text automatically generated by the fMRIprep software) is provided in Supplementary Materials. Further details of the preprocessing pipeline can be found in fMRIPrep’s documentation (https://fmriprep.org/en/latest/workflows.html).

#### 2.7.1 Anatomical data preprocessing

For each participant, the T1-weighted (T1w) image was corrected for intensity non-uniformity and used as T1w-reference throughout the workflow. The T1w-reference was then skull-stripped and brain tissue segmentation of cerebrospinal fluid (CSF), white-matter (WM), and gray-matter (GM) was performed on the brain-extracted T1w image. Volume-based spatial normalisation to the MNI152NLin6Asym standard space was performed through nonlinear registration, using brain-extracted versions of both T1w reference and the T1w template.

#### 2.7.2 Functional data preprocessing

For each of the BOLD runs found per participant (across all tasks and sessions), the following preprocessing was performed. First, a reference volume and its skull-stripped version were generated using a custom methodology of fMRIPrep. Head-motion parameters with respect to the BOLD reference (transformation matrices, and six corresponding rotation and translation parameters) were estimated before any spatiotemporal filtering. The BOLD time-series^2^ were resampled onto their original, native space by applying the transforms to correct for head-motion (No slice-timing correction was applied.). The BOLD reference was then co-registered to the T1w reference. Confounding time-series were then calculated (see below). Finally, the BOLD time-series were resampled into the MNI152NLin6Asym standard space.

fMRIPrep calculates several confounding time-series based on the preprocessed BOLD. Of these, we selected standardised DVARS (a measure of how much image intensity has changed across image frames) as a confound regressor in our design matrix, and we used framewise displacement (FD) as a criterion for excluding scans with excessive movement (see *fMRI statistical analysis*, below). fMRIPrep also extracts a set of physiological regressors to allow for component-based noise correction (CompCor; Behzadi et al., 2007). Principal components are estimated after high-pass filtering the preprocessed BOLD time-series (using a discrete cosine filter with 128 s cut-off) for the two CompCor variants: temporal (tCompCor) and anatomical (aCompCor). We used a selection of the aCompCor regressors in our design matrix (see below). For aCompCor, three probabilistic masks (CSF, WM, and combined CSF+WM) are generated in anatomical space. For each CompCor decomposition, the k components with the largest singular values are retained, such that the retained components’ time-series are sufficient to explain 50% of variance across the nuisance mask (CSF, WM, combined, or temporal). The remaining components are dropped from consideration. Another set of confound regressors calculated by fMRIPrep that we used in our design matrix comprised the head-motion estimates and their temporal derivatives and quadratic terms (Satterthwaite et al., 2013).

### 2.8 fMRI statistical analysis

The imaging data were processed and analysed using the fMRI Feat Analysis Tool (FEAT) Version 6.00 of FSL, part of the software library of the Oxford Centre for Functional MRI of the Brain (fMRIB; https://fsl.fmrib.ox.ac.uk/fsl/fslwiki/). At the first level of analysis, the following pre-statistics processing was applied: non-brain removal using BET (Smith, 2002); spatial smoothing using a Gaussian kernel of FWHM 6.0 mm; grand-mean intensity normalisation of the entire 4D dataset by a single multiplicative factor; and high-pass temporal filtering (Gaussian-weighted least-squares straight line fitting, with sigma = 125 s, i.e., a high-pass filter cut-off of 250 s). Time-series statistical analysis was carried out using FILM with local autocorrelation correction (Woolrich et al., 2001).

The design matrix for this block-design study included 6 stimulus-condition vectors, whose elements represent the onsets and durations of the stimulus blocks (box-car function) convolved with the hemodynamic response function, modelled with the double gamma HRF function (phase = 0 s). The 6 stimulus-condition regressors were: facing communicative interactions, facing individual actions, non-facing communicative interactions, non-facing individual actions, scrambled facing communicative interactions, and scrambled individual facing actions. Besides the stimulus regressors, the design matrix also included confounding regressors calculated via fMRIPrep. We selected 24 head-motion parameters (the 3 translation and 3 rotation parameters, the square of the 6 motion parameters and their temporal derivatives), the first 6 anatomical component-based noise correction (aCompCor) components, and standard deviation of DVARS. Furthermore, rather than select the option in FSL to apply temporal filtering directly to the model, we instead included in the model the first 4 cosine regressors to account for low-frequency confounding signals (fMRIprep produced 7 cosine regressors for our data; the first 4 of these cosine regressors together were equivalent to the high-pass filter applied to our data, i.e., with a cut-off of 250 s.). We further modelled out volumes with extensive motion (i.e., scrubbing) by adding a single time-point nuisance regressor for each volume with framewise displacement >0.9 (an arbitrary threshold meant to serve as a relatively high threshold for motion exclusion, as recommended by Siegel et al., 2014). As noted above, one participant had >10% scrubbed volumes for each of 2 imaging runs, and so we decided to exclude those 2 runs. For the final analysed dataset, there was an average of 0.67% scrubbed volumes per run (SD = 1.33%, range: 0-6.54%).

At the second level (i.e., the level at which the parameter and contrast estimates from the first level are averaged across runs within each participant), analysis was carried out using a fixed-effects model, by forcing the random effects variance to zero in FLAME (FMRIB’s Local Analysis of Mixed Effects) (Beckmann et al., 2003; Woolrich, 2008; Woolrich et al., 2004). At the third (i.e., group) level, analyses were carried out using FLAME mixed-effects stages 1 + 2, over the contrast maps derived from the second-level analysis and with participant as a random effect. *Z* (Gaussianised T/F) statistic images were thresholded using clusters determined by *Z* > 3.1 and a (corrected) cluster significance threshold of *p* = .05 (Worsley, 2001). For display, the thresholded statistical maps were overlaid on the mean high-resolution structural image for the 33 participants, using FSLeyes (FSL’s image viewer). Activated regions were labelled using a combination of the Harvard-Oxford Cortical Structural Atlas (Desikan et al., 2006) and the Julich-Brain Cytoarchitectonic Probabilistic Atlas (Eickhoff et al., 2005, 2006, 2007).

## 3 Results

### 3.1 Behavioural performance

On each trial, participants judged whether the two crosses had the *same* or *different* brightness. Table 1 presents the mean accuracy (proportion correct) and reaction times (s) in the different conditions averaged across participants. Overall, there were no differences in task performance across the different conditions. The accuracy and reaction-time data were submitted to a 2 (stimulus type: communicative, individual) x 3 (stimulus condition: facing, nonfacing, scrambled) analysis of variance (ANOVA). There were no significant main effects of stimulus type (accuracy: *p* = .301, reaction time: *p* = .403) or stimulus condition (accuracy: *p* = .064, reaction time: *p* = .236), nor a significant interaction between the two factors (accuracy: *p* = .065, reaction time: *p* = .537). We also compared each of the coherent conditions to its corresponding scrambled condition using a paired-samples *t*-test. There was a trend for a significant difference in accuracy for the communicative-reversed actions (*p* = .012), which was not significant after Bonferroni correction for multiple post hoc comparisons. The *p* values for all other comparisons were: *p*s > .144.

**Table 1.** Mean accuracy (proportion correct) and reaction times for discriminating the brightness of the two fixation crosses that were overlaid on the point-light figures, as a function of action type and facing (SDs in brackets).

	Communicative			Individual		
	Facing	Nonfacing	Scrambled	Facing	Nonfacing	Scrambled
Accuracy	0.93 (0.02)	0.95 (0.02)	0.92 (0.02)	0.94 (0.02)	0.94 (0.02)	0.94 (0.02)
Reaction times (s)	1.34 (0.05)	1.37 (0.05)	1.33 (0.06)	1.38 (0.06)	1.37 (0.05)	1.33 (0.05)

### 3.2 Imaging results

#### 3.2.1 Brain regions sensitive to communicative interactions

**Fig. 3.**
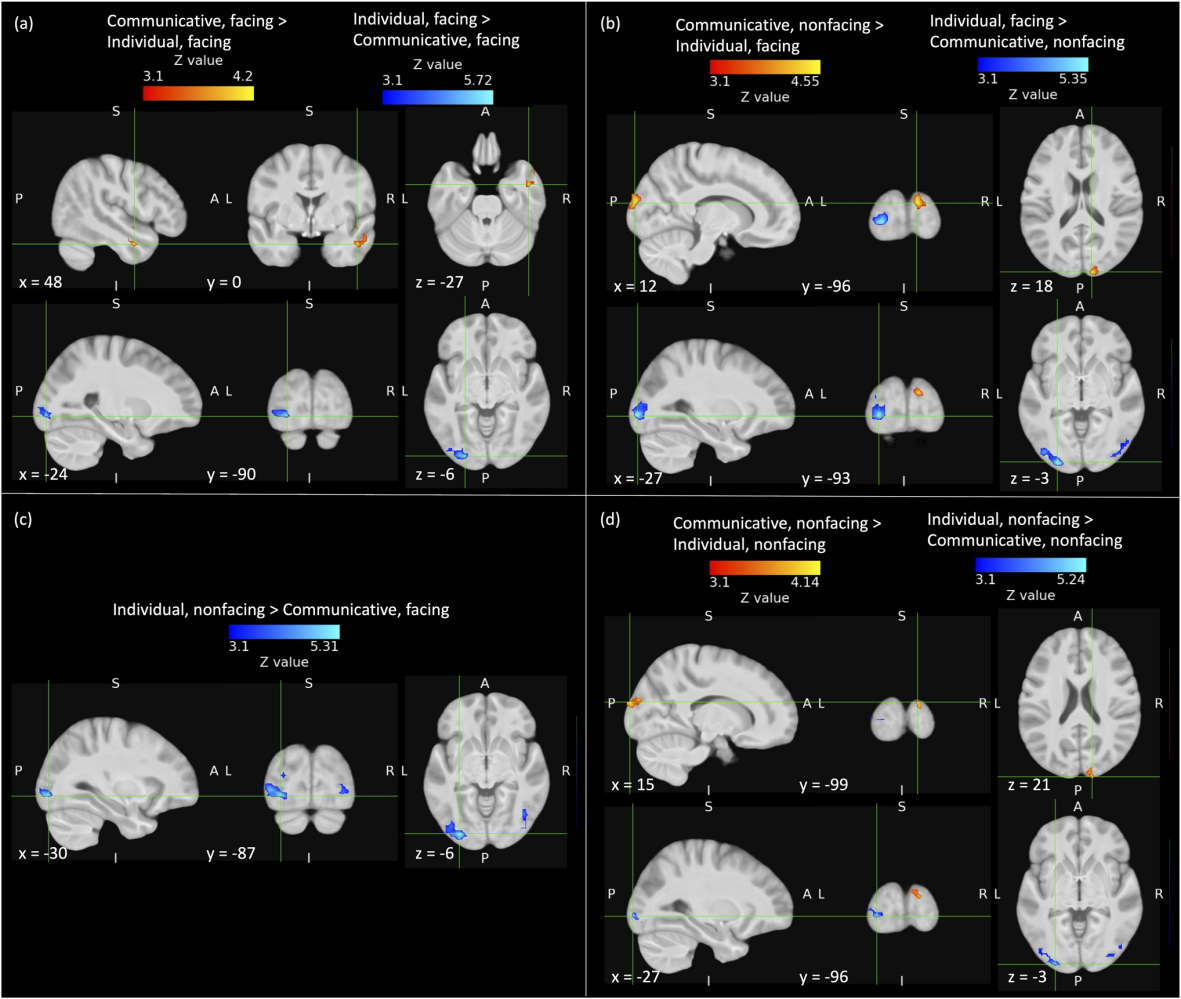
Cortical regions activated for incidental processing of communicative vs. individual actions: (a) communicative interactions (facing only) vs. individual actions (facing only); (b) communicative interactions (nonfacing only) vs. individual actions (facing only); (c) communicative interactions (facing only) vs. individual actions (nonfacing only); (d) communicative interactions (nonfacing only) vs. individual actions (nonfacing only). Hot colours show regions activated by communicative > individual actions, cold colours show regions activated by individual > communicative actions. Images were thresholded using clusters determined by *Z* > 3.1 and a corrected cluster significance threshold of *p* = .05 across the whole brain. No activations for the contrast communicative (facing only) > individual (nonfacing only) survived correction for multiple comparisons. The crosshairs mark the largest *Z* value for each contrast (see Tables 2 & 3). A = anterior, P = posterior, S = superior, I = inferior, L = left, R = right.

We first tested for the effects of stimulus type (communicative vs. individual) to address our main research question. Of particular interest were the effects of our stimulus manipulations (facing direction and point-light dot scrambling) on the comparison of communicative interactions with individual actions. The main effect of stimulus type collapsed across facing direction revealed activation in early visual cortices, namely, in V2 and V3d (Table 2). The facing direction manipulation was implemented to reveal brain areas sensitive to differences in the spatiotemporal contingencies between communicative interactions and individual actions. The significant activation in V2 and V3d was driven by the reversal of the facing direction of the gesturing agent in the communicative interactions only, that is, it was evident for the contrasts of nonfacing communicative interactions with both facing and nonfacing individual actions (Fig. 3b & 3d), but not for facing communicative interactions compared to either facing or nonfacing independent actions. Instead, the contrast of facing communicative interactions > facing independent actions revealed significant activation in right anterior STS (Fig. 3a, Table 2), whereas the contrast of facing communicative interactions > nonfacing individual actions did not produce any significant activations. The reverse contrasts, testing the main effect of individual actions > communicative interactions and the simple main effects constituting the factorial combinations of facing direction with action type, revealed significant clusters of activation in bilateral lateral occipital cortex and occipital pole (Fig. 3, Table 3).

**Table 2.** Significant clusters for the whole-brain contrasts of communicative interactions > individual actions.

Hemisphere	Brain regions	MNI coordinates			Max *Z* value	Cluster *p* value	Size (# voxels)
		*x*	*y*	*z*			
*Communicative interactions, facing + nonfacing > Individual actions, facing + nonfacing*							
R	Occipital pole: areas hOc2 (V2), hOc3d (V3d)	12	-96	18	4.7	.00012	103
*Communicative interactions, facing only > Individual actions, facing only* (Fig. 3a)							
R	Middle temporal gyrus/anterior STS: area TE 5	48	0	-27	4.15	.00035	75
*Communicative interactions, nonfacing only > Individual actions, facing only* (Fig. 3b)							
R	Occipital pole: areas hOc2 (V2), hOc3d (V3d)	12	-96	18	4.51	.00269	55
*Communicative interactions, nonfacing only > Individual actions, nonfacing only* (Fig. 3d)							
R	Occipital pole: areas hOc2 (V2), hOc3d (V3d)	15	-99	21	4.1	.0411	37
*(Communicative, facing - Scrambled communicative) > (Individual, facing - Scrambled individual)* (Fig. 4)							
R	Brain stem & cerebellum (V & VI)	12	-30	-36	4.04	<.0001	123
L	Precentral & cingulate gyri: areas 5 M (SPL) & 5Ci (SPL)	-9	-33	48	4.06	.00016	83
L	Amygdala (centromedial & basolateral), basal forebrain (area CH4)	-24	-6	-9	4.12	.00465	50
L	Supramarginal gyrus/inferior parietal lobule: area PFt	-54	-33	42	3.96	.0458	31

MNI coordinates and *Z* values are for the peak in each cluster; cluster size is for the unthresholded map.

**Table 3.** Significant clusters for the whole-brain contrasts of individual actions > communicative interactions.

Hemisphere	Brain regions	MNI coordinates			Max *Z* value	Cluster *p* value	Size (# voxels)
		*x*	*y*	*z*			
*Individual actions, facing + nonfacing > Communicative interactions, facing + nonfacing*							
L	Lateral occipital cortex & occipital pole: areas hOc3v (V3v), hOc4lp, hOc4v (V4v)	-30	-90	-6	5.76	<.0001	346
R	Lateral occipital cortex: areas hOc4la, hOc5 (V5/MT)	45	-75	3	4.95	<.0001	300
*Individual actions, facing > Communicative interactions, facing* (Fig. 3a)							
L	Lateral occipital cortex & occipital pole: areas hOc3v (V3v), hOc4v (V4v), hOc4lp	-24	-90	-6	5.67	<.0001	138
*Individual actions, nonfacing > Communicative interactions, facing* (Fig. 3c)							
L	Lateral/inferior occipital cortex: areas hOc4lp, hOc4v (V4v), hOc3v (V3v)	-30	-87	-6	5.25	<.0001	188
R	Lateral/inferior occipital cortex: areas hOc4lp, hOc4v (V4v), hOc5 (V5)	33	-87	-3	4.34	<.0001	150
*Individual actions, facing > Communicative interactions, nonfacing* (Fig. 3b)							
L	Occipital pole/lateral occipital cortex: areas hOc4lp, hOc3v (V3v), hOc4la	-27	-93	-3	5.29	<.0001	177
R	Lateral occipital cortex: areas hOc4la, hOc5 (V5/MT)	54	-69	3	4.41	<.0001	139
*Individual actions, nonfacing > Communicative interactions, nonfacing* (Fig. 3d)							
L	Occipital pole/lateral occipital cortex: areas hOc4lp, hOc3d (V3d), hOc3v (V3v)	-27	-96	-3	5.18	.00169	70
R	Lateral occipital cortex/occipital pole: areas hOc4lp, hOc4v (V4v)	36	-87	-3	3.83	.0198	44

MNI coordinates and *Z* values are for the peak in each cluster.

**Fig. 4.**
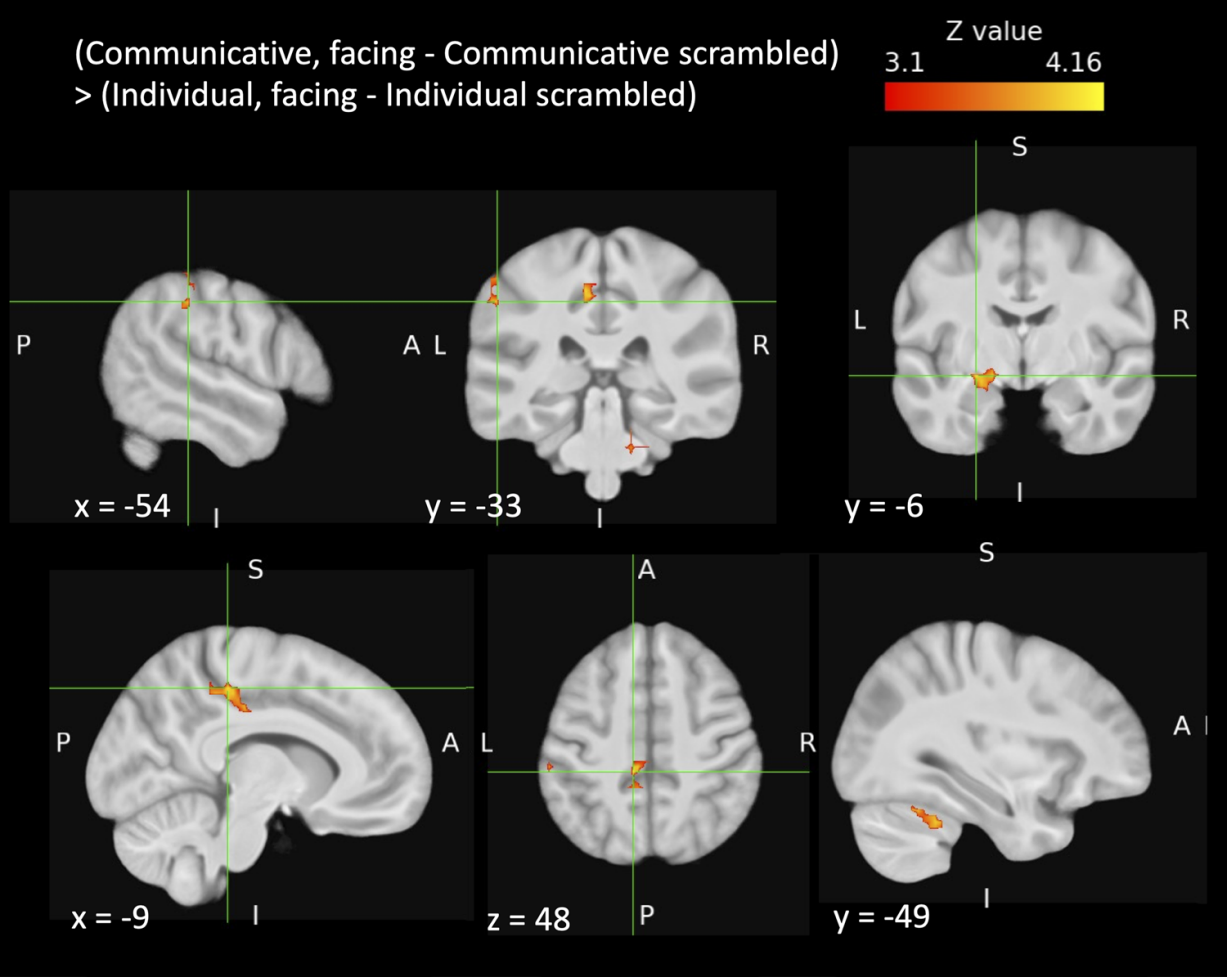
Cortical regions activated for incidental processing of communicative actions > individual interactions, after subtraction of their scrambled counterparts. The crosshairs mark the largest *Z* value for each cluster (see Table 2). Top row: cluster in left supramarginal gyrus shown in the left and middle images and cluster in left amygdala shown in the right image. Bottom row: precentral/cingulate gyrus cluster shown in the left and middle images, and the cerebellum cluster (whose peak is in the brainstem) shown in the right image. Images were thresholded using clusters determined by *Z* > 3.1 and a corrected cluster significance threshold of *p* = .05 across the whole brain. A = anterior, P = posterior, S = superior, I = inferior, L = left, R = right.

The spatial scrambling of the point-light dots allowed us to test the contrast of communicative interactions > individual actions after having subtracted out the respective contributions of the local dot motions in each of these two types of dyadic action displays. This contrast was implemented to reveal brain areas sensitive to differences in global motion and particularly motion-mediated structural information in the communicative interactions compared to the individual action displays. This contrast—namely (communicative facing - scrambled communicative) > (individual facing - scrambled individual)—revealed significant clusters in brain stem/cerebellum, left amygdala, left inferior parietal lobule (supramarginal gyrus), and left medial superior parietal lobule (precentral/cingulate gyri) (Fig. 4, Table 2). The reverse contrast—namely (individual facing - scrambled individual) > (communicative facing - scrambled communicative)—did not reveal any significant clusters of activation.

#### 3.2.2 Role of posterior STS in communicative interactions

It is notable that right posterior STS was not one of the regions activated by communicative interactions compared to individual actions in any of the relevant contrasts, given numerous previous findings of communicating or otherwise socially interacting dyads eliciting posterior STS activation (Centelles et al., 2011; Georgescu et al., 2014; Isik et al., 2017; Landsiedel et al., 2022; Sapey-Triomphe et al., 2017; Walbrin et al., 2018, 2020). In view of this previous evidence, we conducted further analyses focused on posterior STS to determine whether this region played a role when participants incidentally processed communicative interactions.

**Fig. 5.**
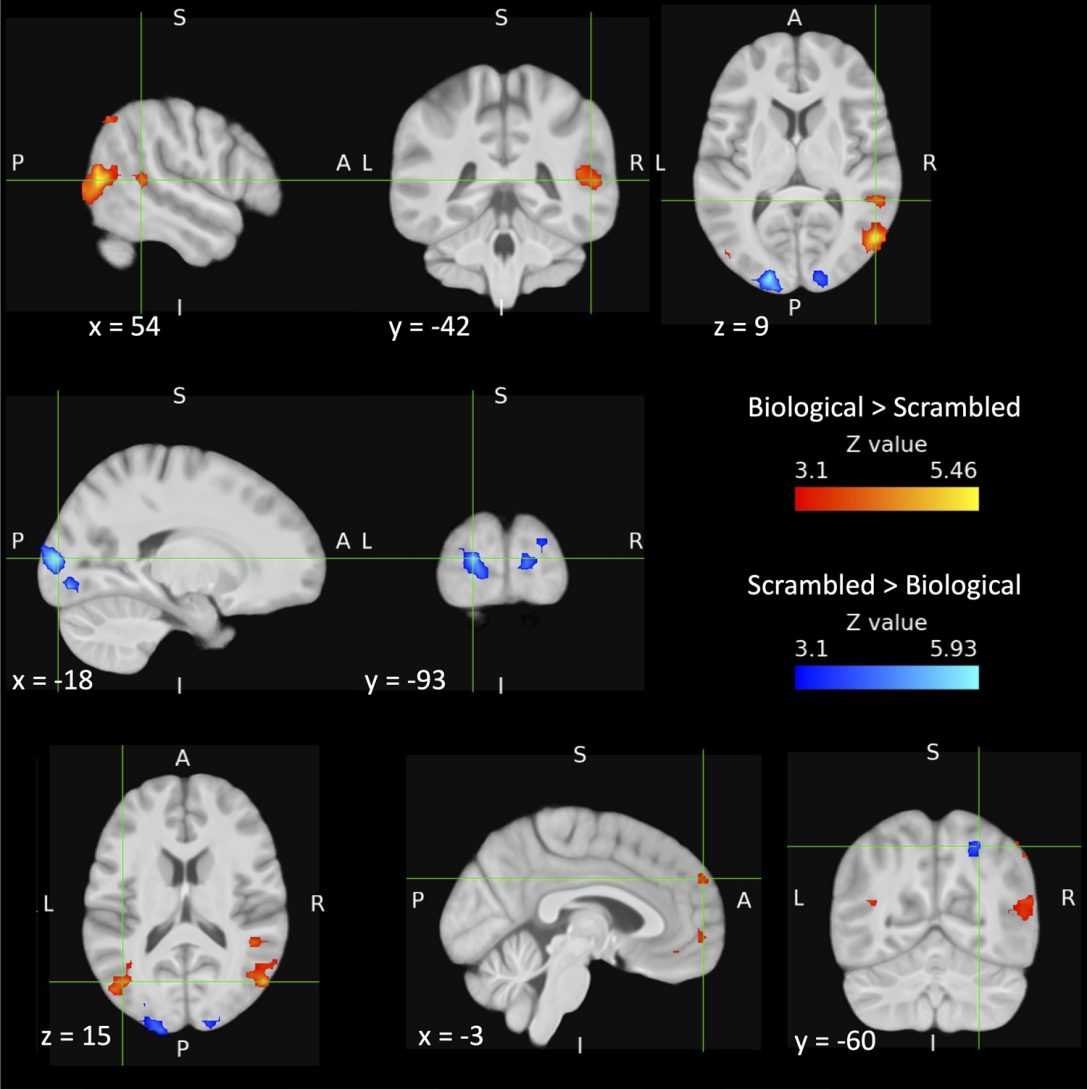
Cortical regions activated for the incidental processing of biological vs. scrambled point-light motion. Hot colours show regions activated by biological motion > scrambled point-light motion, cold colours show regions activated by scrambled point-light motion > biological motion. The crosshairs mark the largest *Z* value in the right STS cluster (top row), the occipital pole (V3d, V2) cluster (middle row), and in the clusters in left lateral occipital cortex (V5, PGp & PGa), superior frontal gyrus, and right lateral occipital/superior parietal cortex (bottom row, left to right). Images were thresholded using clusters determined by *Z* > 3.1 and a corrected cluster significance threshold of *p* = .05. The biological motion condition consisted of the stimuli from all 4 main experimental conditions (communicative interactions and individual actions, and their nonfacing counterparts). The scrambled point-light motion condition consisted of spatially scrambled versions of the facing communicative interactions and individual actions. A = anterior, P = posterior, S = superior, I = inferior, L = left, R = right.

We first tested for areas responsive to point-light biological motion relative to scrambled point-light motion. This contrast revealed significant BOLD responses in bilateral posterior STS and lateral occipital cortex, with larger and more extensive activations in the right hemisphere, as well as clusters in medial prefrontal cortex (anterior cingulate) and posterior cingulate (Fig. 5, Table 4). This finding gave us confidence in the stimuli, task, and experimental design, given the considerable previous fMRI evidence of point-light biological motion relative to scrambled point-light motion eliciting activation in posterior STS and lateral occipital cortex (e.g., Alaerts et al., 2017; Anderson et al., 2013; Grossman et al., 2000; Jastorff & Orban, 2009; Peuskens et al., 2005; Saygin et al., 2004).

**Table 4.** Significant clusters for the whole-brain contrasts of biological vs. scrambled point-light motion.

Hemisphere	Brain regions	MNI coordinates			Max *Z* value	Cluster *p* value	Size (# voxels)
		*x*	*y*	*z*			
*Biological motion > Scrambled point-light motion* (Fig. 5)							
R	Lateral occipital cortex: areas hOc5 (V5), inferior parietal lobule (PGp & PGa)	54	-69	9	5.41	<.0001	244
L	Lateral occipital cortex: areas hOc5 (V5), inferior parietal lobule (PGp & PGa)	-42	-69	15	5.11	<.0001	115
R	Superior temporal sulcus	54	-42	9	4.31	.00095	64
L	Superior frontal gyrus/frontal pole	-3	57	33	3.83	.00876	44
R	Lateral occipital cortex/inferior parietal lobule: areas PGa, PGp	51	-63	48	4.35	.0263	35
L	Frontal pole: areas Fp2, p32	-9	57	0	3.92	.0339	33
*Scrambled > Biological point-light motion* (Fig. 5)							
L	Occipital pole: areas hOc3d (V3d), hOc2 (V2)	-18	-93	9	5.87	<.0001	180
R	Lingual & occipital fusiform gyri: areas hOc2 (V2), hOc3v (V3v)	15	-84	-6	4.80	<.0001	131
R	Lateral occipital cortex/superior parietal lobule	27	-60	51	4.08	.00349	52

MNI coordinates and *Z* values are for the peak in each cluster.

We next focused our analyses on posterior STS. In our first analysis, we performed the same univariate analysis for the communicative versus individual contrast but this time over a more restricted area of cortex, namely, those regions activated by our group-level orthogonal contrast of biological > scrambled point-light motion (i.e., to the bilateral posterior STS, lateral occipital, and medial prefrontal regions shown in Fig. 5). Even with this more focused analysis (i.e., inclusively masked by the thresholded biological > scrambled contrast), none of the contrasts of communicative interactions with individual actions revealed any significant activation (voxelwise correction, *p* < .05). It is important here to note that the coordinates of the group activation peaks in posterior STS recorded for interacting versus independently acting dyads in several previous studies all fall within the cluster defined by the biological motion > scrambled motion contrast in the present study^3^.

Given these potential limitations, we conducted three sets of region-of-interest (ROI) analyses on right posterior STS. For one set, subject-specific ROIs consisted of the voxels within a 6 mm-radius of the subject-specific peak in right posterior STS for the orthogonal contrast biological > scrambled point-light motion that survived a threshold of *p* < .05 uncorrected. For the other two sets, ROIs consisted of voxels within a 6 mm-radius sphere centred at the group coordinates for the peak of the activation in right STS reported by either Isik et al. (2017) or Walbrin et al. (2018) for the contrast communicative interactions > individual actions. These two studies were chosen because the relevant coordinates were within the right STS cluster from our own group contrast of biological > scrambled point-light motion and because the stimuli in both studies were drawn from the same database as were our stimuli; see below for further discussion of this latter point. For each individual ROI analysis, we calculated the mean subject-specific *Z* value across all voxels within the ROI for the relevant contrast (using the “fslmeants” function in FSL) and subjected these mean *Z* values to a one-tailed, one-sample *t*-test against a value of 0. For each set of ROI analyses, we tested 5 contrasts, as listed in Table 5. For the posterior STS ROI centred on the subject-specific peaks for biological > scrambled motion, the contrast (communicative facing - scrambled communicative) > (individual facing - scrambled individual) showed that the mean *Z* score (*M* = .35, SD = 0.74) was significantly greater than 0 prior to correction for multiple comparisons (*p* = .011), with a small-to-medium effect size (*d* = 0.47), but not following correction (for 5 comparisons, corrected *α* = .01; for all 15 comparisons, corrected *α* = .0033). As can be seen from Table 5, none of these ROI analyses returned a significant result.

**Table 5.** Summary results for one-sample *t*-tests on mean *Z* scores for right posterior STS ROIs.

Contrast	*t* Value	***p*** Value	Effect size (***d***)	95% CI
*Right pSTS ROI centred on subject-specific peak for biological > scrambled motion* (N = 27)				
Communicative facing > individual facing	1.76	.045	0.34	(0.01, ∞)
Communicative facing > individual non-facing	0.72	.24	0.14	(-0.18, ∞)
Communicative non-facing > individual facing	-1.05	.85	-0.2	(-0.52, ∞)
Communicative non-facing > individual non-facing	-1.9	.97	-0.37	(-0.69, ∞)
(Communicative facing - scrambled communicative) > (individual facing - scrambled individual)	2.43	.011	0.47	(0.13, ∞)
*Right pSTS ROI centred on coordinates from Isik et al. (2017)* (N = 33)				
Communicative facing > individual facing	1.11	.14	0.19	(-0.1, ∞)
Communicative facing > individual non-facing	0.82	.21	0.14	(-0.15, ∞)
Communicative non-facing > individual facing	0.0	.5	0.0	(-0.29, ∞)
Communicative non-facing > individual non-facing	-0.31	.62	-0.05	(-0.34, ∞)
(Communicative facing - scrambled communicative) > (individual facing - scrambled individual)	1.59	.06	0.16	(-0.13, ∞)
*Right pSTS ROI centred on coordinates from Walbrin et al. (2018)* (N = 33)				
Communicative facing > individual facing	1.59	.06	0.28	(-0.02, ∞)
Communicative facing > individual non-facing	0.53	.3	0.09	(-0.2, ∞)
Communicative non-facing > individual facing	0.84	.21	0.15	(-0.14, ∞)
Communicative non-facing > individual non-facing	-0.33	.63	-0.06	(-0.34, ∞)
(Communicative facing - scrambled communicative) > (individual facing - scrambled individual)	1.63	.06	0.28	(-0.01, ∞)

Note: All ROIs were defined by a 6 mm-radius sphere centred at the relevant coordinates. The ROIs centred on subject-specific peaks for biological > scrambled motion consisted of only those voxels within the sphere that passed the threshold of *p* < .05 uncorrected. The ROIs centred on the coordinates from Isik et al. (2017) and Walbrin et al. (2018) consisted of all voxels within the sphere.

## 4 Discussion

Here, we aimed to identify brain regions involved in the incidental processing of communicative interactions in visual scenes containing only bodily movements. We defined incidental processing as the processing of the stimuli outside the participant’s task-related focus of attention. This was achieved by having participants perform a task requiring visual attention to the location of two interacting or independently acting agents but not to the agents themselves (a same-different judgement on the brightness of two fixation crosses on the screen). Two stimulus manipulations were implemented, which we used to reveal brain regions involved in processing different visual cues underpinning incidental processing of communicative interactions. By reversing the facing direction of one of the agents, we could test for brain regions sensitive to the spatiotemporal contingencies of the two agents. By including versions of the point-light actions in which the vertical starting positions of the point-lights were scrambled, we could set up a contrast in which we compared the activation elicited by communicative interactions versus independent actions after having subtracted out the contributions of their respective local dot motions, thus testing for brain regions sensitive to differences in visual cues related to global motion, including structure-from-motion information. In what follows, we first briefly summarise the main findings and then discuss in more detail each of them in turn.

### 4.1 Summary of main findings

Point-light displays of communicative interactions elicited activation in right anterior temporal cortex (on and around STS) when the two agents were facing each other, relative to the individual actions (i.e., for the contrast communicative facing > individual facing). By comparison, when one of the agents in the communicative or independent actions faced away from the other agent (communicative nonfacing > individual facing/nonfacing), we found activity concentrated instead in the early right visual cortex (V2 and V3d). Given that this stimulus manipulation disrupts spatiotemporal contingencies between the two agents because of the spatial change in facing direction, we infer that the right anterior temporal cortex/STS is sensitive to these spatiotemporal contingencies. Independently acting dyads, on the other hand, activated regions of the lateral occipital cortex and the occipital pole bilaterally, compared to communicative interactions, regardless of facing direction (i.e., for the contrasts individual facing/nonfacing > communication facing/nonfacing).

Controlling for differences in the local motion of the point-light dots between the communicative interactions and independent actions revealed significant activation in left inferior parietal lobule (supramarginal gyrus), left medial superior parietal lobule (precentral/cingulate gyri), brain stem/right cerebellum, and left amygdala. Independent actions compared to communicative interactions, on the other hand, did not significantly activate any regions after controlling for differences in local motion. Thus, for communicative interactions, facing direction and global structure-from-motion (i.e., scrambling manipulation) affected the regions that were involved in their incidental processing. These manipulations did not influence activation to individual actions: Overall, regions in the early visual cortex responded more to individual actions (compared to communicative ones), irrespective of facing direction and irrespective of subtracting out local motion cues. We further note that any differences between stimulus types and conditions are not due to response differences, as participants performed equally accurately and quickly on the brightness-discrimination task across all 6 task blocks.

Lastly, it is notable that we did not find any evidence for the involvement of posterior STS or body-selective EBA in the incidental processing of communicative interactions compared to independent actions, despite previous research strongly implicating these regions in the visual processing of third-person social interactions when participants passively view or explicitly evaluate such stimuli (Abassi & Papeo, 2020, 2022; Centelles et al., 2011; Gandolfo et al., 2023; Georgescu et al., 2014; Isik et al., 2017; Landsiedel et al., 2022; Walbrin & Koldewyn, 2019; Walbrin et al., 2018, 2020). Given that that the absence of evidence does not imply evidence of absence, it will be important for future research to test this further.

### 4.2 Brain regions for incidental processing of social interactions

A few previous studies have reported evidence of anterior temporal lobe involvement in the processing of whole-body movements of *single* individuals. Vaina et al. (2001) reported activation in the left (but not right) anterior temporal lobe when participants discriminated biological motion but not when they discriminated non-rigid motion, even though identical stimuli were used in both tasks, suggesting that such anterior temporal lobe activation for biological motion *per se* depends on the task-related focus of attention (i.e., that incidental processing of biological motion does not recruit anterior temporal cortex). Moreover, Vaina and Gross (2004) demonstrated that lesions to the right anterior temporal lobe are associated with impairments in recognising biological motion. More directly relevant to the present study, the right anterior temporal cortex (temporal pole) in the monkey was one of several regions responsive to interactions between two monkeys compared to physical interactions between two objects, in Sliwa and Freiwald’s (2017) fMRI study. Even more directly relevant are the findings of two fMRI studies with humans. Centelles et al. (2011) reported that social interactions versus independent actions in point-light displays activated, amongst other areas, a region of the right anterior temporal cortex/STS, which overlaps with the activation we report for social interactions versus independent actions when both agents were facing each other. Sapey-Triomphe et al. (2017) also reported activation in the right anterior temporal lobe/ STS (amongst other regions) for social interactions versus independent actions in point-light and stick-light^4^ displays, not only in adult participants but also in adolescents and children, again overlapping the anterior STS activation reported in the present study. Yet the participants in both Sapey-Triomphe et al.’s and Centelles et al.’s studies made explicit judgements as to whether the agents were interacting or not, whereas our participants performed an orthogonal task (one that was not directed at the nature of the body movements).

Other studies that have used passive viewing, in which participants were not explicitly required to judge the type of actions, found evidence for the anterior temporal cortex/STS activation to social stimuli (Isik et al., 2017; Walbrin et al., 2018, 2020, 2023). Interestingly, they also found that this region showed activation to social interactions when using full-body videos (Walbrin & Koldewyn, 2019). These studies, however, could not distinguish the visual features that were used for the incidental processing of social interactions. By comparison, we found that the right anterior temporal cortex/STS activation for communicative interactions compared to independent actions only when the agents faced each other, which highlights the importance of spatial *and* temporal contingencies between the two agents. This is because the spatial change realised by reversing the facing direction of the agent issuing the communicative instruction disrupts those spatiotemporal contingencies more when that agent is paired with an agent who responds to that instruction than with an agent who acts independently of the instructing gesture. This interpretation of the right anterior STS’s role in the visual perception of social interactions is consistent with neurophysiological data from monkeys: The right and left anterior temporal sulci of the monkey contain not only cells selectively responsive to whole-body movements or the movements of individual limbs (e.g., Perrett et al., 1985), but also cells selectively responsive to the orientation of the body, its direction of motion, or to the specific type of body motion (Oram & Perrett, 1994), and cells selectively responsive to specific combinations of body form and motion direction (Oram & Perrett, 1996). Nonetheless, neuroimaging and neurostimulation evidence from studies using single-body stimuli shows that the regions of human STS (and occipitotemporal cortex more generally) that separately represent and integrate the form and motion of bodies are located in more regions posterior to the anterior temporal cortex (e.g., Jastorff & Orban, 2009; Jastorff et al., 2016; Vangeneugden et al., 2014).

We also found that communicative interactions compared to individual actions elicited activation in two regions of the left parietal lobe, but only in the contrast in which we subtracted out activity in each condition due to the local motion of the point-light dots. This finding suggests that the incidental processing of communicative interactions may involve structure-from-motion mechanisms carried out by the parietal lobe (e.g., Peuskens et al., 2004). The medial cluster spanned areas 5M and 5Ci of the superior parietal lobule. The lateral cluster was in the inferior parietal lobule, centred in area PFt (on supramarginal gyrus) and bordering area PF and anterior intraparietal sulcus. We now discuss each of these findings in turn.

Both Isik et al. (2017) and Walbrin et al. (2023) also reported activation (in adult participants) in the medial parietal cortex for communicative interactions compared to independent actions, which was much more extensive and stronger in the left than in the right hemisphere in Walbrin et al.’s study, using point-light communicative interactions from the same database that we used for our stimuli (Manera et al., 2015). However, neither study reports more precise anatomical locations of those activations, nor the coordinates of those activations, so it is not possible to check whether those activations overlap with the medial parietal activation reported here; nonetheless, visual inspection of the left hemisphere activation in Walbrin et al.’s study strongly suggests some overlap. Moreover, the stimuli used in these two previous studies—as well as in other studies by Walbrin et al. (2018, 2020)—differed from ours in four key respects. First, the stimuli comprising the independent actions in these other studies were obtained from a different database (Vanrie & Verfaillie, 2004) so there may be unknown stimulus differences in how the actions were initially motion-captured (although both sets were created by the same lab). Second, the independent action stimuli in these other studies had a white line drawn between the two agents to increase the impression that they were acting independently, whereas this was not the case in our stimuli. Third, none of the studies from this group reversed the facing direction for the communicative actions, again leading to potential low-level stimulus differences between communicative and individual point-light dyads. Our stimuli were thus constructed and manipulated specifically to test for brain regions sensitive to differences in the spatiotemporal contingencies between interacting and independently acting dyads. Fourth and lastly, participants in these other studies passively viewed the body movement stimuli, whereas our participants engaged in a task unrelated to those stimuli.

Activations in the left inferior parietal cortex, particularly in and around the intraparietal sulcus, are frequently reported in response to point-light biological motion stimuli as compared to other forms of coherent motion or scrambled motion (e.g., Alaerts et al., 2017; Grèzes et al., 2001; Koldewyn et al., 2011; Peuskens et al., 2005). Bilateral intraparietal sulcus activation has also been reported for social interactions versus independent actions in point-light displays (Centelles et al., 2011). Notably, activation in the left intraparietal sulcus and neighbouring regions of the inferior parietal lobule has been reported for contingent compared to mirrored actions in full-body avatar dyads (Georgescu et al., 2014). FMRI evidence indicates that the inferior parietal lobule (IPL) encodes observed goal-directed actions according to the type of action, regardless of the effector (hand, foot, mouth) used (Jastorff et al., 2010). Area PFt, in particular, is an established part of the action observation and imitation network (e.g., Caspers et al., 2010; Passingham et al., 2014; Van Overwalle & Baetens, 2009) and its equivalent area in macaque monkeys (PF) is part of the mirror neuron system (e.g., Caspers et al., 2011; Rizzolatti & Craighero, 2004). Interestingly, a recent fMRI study found evidence that area PFt and the anterior portion of the anterior intraparietal sulcus are activated more by observation of (videos of) one person performing indirect communicative actions than it is by observation of either two people engaging in direct communicative actions or one person manipulating an object (Urgen & Orban, 2021) (Indirect communications were defined as actions by a single individual that consist of leaving a symbolic trace, such as a message or shape, in a substrate that can be viewed later by another individual.). Indeed, using multivoxel pattern analysis and representational similarity analysis, this study provided evidence that a region of PFt represents indirect communicative actions. Yet direct communicative actions did not activate any region more than indirect communicative actions or object-manipulative actions in Urgen and Orban’s (2021) study. Crucially, however, the direct communication stimuli in that study consisted of one person performing a communicative gesture and the other person not reacting. Our stimuli, by contrast, involved both people acting (one person performing a communicative gesture and the other person either responding appropriately to that gesture or performing an independent action). Our data therefore show that left PFt is sensitive to spatiotemporal contingencies between the movements of two people, consistent with the aforementioned finding of Georgescu et al. (2014), and particularly to more global motion and structure-from-motion cues.

### 4.3 Additional brain regions for incidental processing of social interactions

We also found that a region of the right cerebellum was activated by communicative interactions compared to independent actions after subtraction of activity due to local dot motion. There are reports of cerebellum activation to whole-body movements of single individuals (Grossman et al., 2000; Jack et al., 2017; Ptito et al., 2003; Sokolov et al., 2012; Vaina et al., 2001). Visual sensitivity to body motion is impaired after lesions of the left lateral cerebellum but remains relatively intact after lesions of the medial cerebellum (Sokolov et al., 2010). Moreover, left lateral cerebellar regions have been shown to have reciprocal effective connectivity with biological-motion sensitive posterior STS during observation of point-light body movement stimuli (Jack et al., 2017; Sokolov et al., 2012), supported by anatomical connections between these two regions (Sokolov et al., 2014). Yet the relevant activation in the present study is in the right anterior cerebellum. This cluster is very close to and partly overlaps two regions of the cerebellum identified in a meta-analysis of functional imaging studies that are associated with a somatomotor network involved in understanding other people’s bodily movements and actions (Van Overwalle et al., 2014). It is also very close to and partly overlaps regions of the right cerebellum activated by passive viewing of point-light body motion compared to scrambled point-light motion in a study by Jack et al. (2017), who also reported activations in regions of the left cerebellum for the same contrast. Our results suggest that this region of the right cerebellum is, like the regions of the medial and lateral parietal cortex discussed above, sensitive to structure-from-motion cues evident in interacting dyads but absent in independently acting dyads, even in the absence of the observer’s task-related attention.

We found that social interactions compared to independent actions also elicited activation in left amygdala, but again only in the contrast in which we subtracted out activity in each condition due to the local motion of the point-light dots. Amygdala activation (bilateral or unilateral) has occasionally been reported for viewing non-emotional whole-body movements compared to non-biological motion, in tasks requiring explicit judgements about the movements (Bonda et al., 1996; Herrington et al., 2011; Ptito et al., 2003). Moreover, one study has reported greater bilateral amygdala activation, and greater effective connectivity between bilateral amygdala and right temporal pole (as well as left and right posterior STS and left fusiform), for biological compared to scrambled point-light motion in females than in males (Anderson et al., 2013). Bilateral amygdala activation has also been reported for photographic images of two people facing each other versus facing away from each other, in a task requiring an explicit judgement about the attitude of the individuals towards each other or their surroundings (Kujala et al., 2012). Amygdala activation has not previously been reported for whole-body third-person social interactions compared to independent actions, however. In the present study, the amygdala activation was evident only after subtraction of activation due to the local motion of the point-lights in each condition. One interpretation of this finding is that the left amygdala has a role in processing aspects of global motion or structure-from-motion present in the interacting dyads but reduced or absent in the independently acting dyads. We favour, instead, an interpretation that appeals to a primary role of the amygdala in coordinating the function of cortical networks to support the evaluation of biologically significant (and thus often socially and emotionally charged) stimuli (e.g., Pessoa, 2010, 2017; Pessoa & Adolphs, 2010). This latter interpretation is consistent with the fact that the activation we observed in this region was centred in the centromedial nucleus of the amygdala and area CH4 of the basal forebrain, the latter of which includes the lateral part of the bed nucleus of the stria terminalis (Zaborszky et al., 2008). The centromedial nucleus and the bed nucleus of the stria terminalis form key parts of a circuit that links them via ascending projections to a wide array of cortical regions, and which helps subserve not only the ability to sustain attention, but also more selective attention functions, including in the visual domain (for a review, see Pessoa, 2010).

Turning the facing direction of the agent issuing the communicative gesture away from the other agent (“follower”) resulted in the now nonfacing communicative interaction stimuli activating right V2 and V3, yet these regions were not significantly activated in the facing communicative > facing individual action contrast, which instead showed activation in right anterior STS. V3 has a role in the processing of second-order motion (Smith et al., 1998) and both V2 and V3 have roles in the extraction of structure-from-motion (Murray et al., 2003; Orban et al., 1999; Paradis et al., 2000; Vanduffel et al., 2002). Recall that our contrast of communicative interactions compared to independent actions after subtraction of activity due to local dot motion was designed to reveal brain regions sensitive to structure-from-motion cues evident in interacting dyads but absent in independently acting dyads. That contrast revealed significant clusters in, amongst other regions, the left parietal cortex but not in V2 or V3, despite the known involvement of V2 and V3 in structure-from-motion processing. These findings are consistent with a distinction between two networks or routes associated with structure-from-motion processing, one involving lower-level visual areas such as V2 and V3 (also present in monkey), and the other involving parietal areas in and around intraparietal sulcus (not evident in monkey) (Orban et al., 2003; Vanduffel et al., 2002).

Compared to communicative interactions, independent actions activated V3v, V4v, hOc4lp, hOc4la, and V5, regardless of agent facing direction, with some hemispheric differences depending on the particular contrast. V3 and V5 have roles in extracting 3D structure-from-motion (Orban et al., 1999; Vanduffel et al., 2002) and V3, especially its ventral portion, has also been implicated in the processing of point-light biological motion possibly because of its role in extracting structure-from-motion (Servos et al., 2002). Areas hOc4la and hOc4lp have roles in shape processing and together constitute most or all of object-selective LOC (Malikovic et al., 2016). V4 is activated by segregated versus uniform textures (Kastner et al., 2000), and V3v and V4v may be specifically involved in segmenting motion information into separate objects (Caplovitz & Tse, 2010), which could explain why we observed activation in these regions for independently acting dyads compared to interacting dyads.

### 4.4 Limitations and future work

We used an orthogonal task (brightness discrimination) that engaged participants on each trial and allowed them to fixate on the location of each point-light display of the dyad on that trial. In contrast to previous studies that used explicit judgement tasks (Centelles et al., 2011; Georgescu et al., 2014; Kujala et al., 2012; Sapey-Triomphe et al., 2017), passive viewing (Isik et al., 2017; Landsiedel et al., 2022; Walbrin & Koldewyn, 2019; Walbrin et al., 2018, 2023, 2020), or rare events occurring at central fixation (Abassi & Papeo, 2020, 2022; Bellot et al., 2021), our task allowed us to determine if communicative interactions, but not individual actions, are incidentally processed. If so, the task further allowed us to identify the visual information used for this processing and the brain regions that may be involved. That said, there are a few limitations in the current study to consider. First, participants were highly accurate (>90%) and responded within a few seconds (~1.30 s). Thus, there may be some opportunities for participants to think about the actions of the dyads. However, as noted in the Introduction, the communicative gestures occur early in the overall communicative interaction. Second, our orthogonal task confined eye movements to fixation changes between relatively small regions of the two fixation crosses and relatedly, restricted foveation of the point-light displays to those locations, as opposed to alternative tasks that would promote wider exploration of the point-light displays. Thus, the role of eye movements for these complex point-light dyads remains unknown for our current studies, as well as for previous studies. Further research is needed to address these limitations. For example, future studies can directly compare implicit and explicit tasks, or use other stimulus manipulations such as stimulus inversion, which can also disrupt configural processing of individual point-light displays (Pavlova & Sokolov, 2000; Sumi, 1984; Troje & Westhoff, 2006). Importantly, future studies can simultaneously track eye movements while acquiring fMRI data to determine the contribution of fixation patterns to brain activations in different regions. Finally, it may also be critical to look at the pattern of connectivity between brain regions when processing communicative versus individual actions (e.g., Walbrin et al., 2023).

### 4.5 Conclusions

The current findings complement existing studies that have delineated the putative brain network involved in processing people interacting with each other. Given the importance of interpreting social interactions between people in many daily situations, we focused on the incidental processing of communicative interactions, in which observers do not need to explicitly make judgements about people’s actions. Importantly, we found that anterior regions of the temporal cortex/STS and parietal cortex are involved in this incidental processing. Our results suggest that additional brain regions may be recruited to process the spatiotemporal contingencies that distinguish these social interactions from people acting individually.

## Data and Code Availability

Neuroimaging data and summary behavioural and stimulus-related data and associated code are available at the project’s OSF page at https://osf.io/nh5w4/.

## Author Contributions

Anthony P. Atkinson: Conceptualisation, Methodology, Investigation, Formal analysis (imaging data), Visualisation (imaging data), Data Curation, Writing—Original Draft, Writing—Review & Editing, and Project administration; Quoc C. Vuong: Conceptualisation, Methodology, Investigation, Software, Formal analysis (stimulus and behavioural data), Visualisation (stimulus data), and Writing—Review & Editing.

## Declaration of Competing Interest

None.

## Supplementary Material

Supplementary Material

## Footnotes

1 In point-light displays, the moving body is represented by a small number of dots against an otherwise plain background, with the dots positioned to highlight the motion of the main body parts (Johansson, 1973, 1975). Thus, static form information is greatly reduced but motion (including form-from-motion) information is preserved. See Figure 2 for examples.

2 Given that we did not have fieldmaps for 7 of the 33 participants, and unsatisfactory results obtained with fMRIPrep’s fieldmap-less susceptibility distortion correction (new distortions were evident in the “corrected” images), we did not perform susceptibility distortion correction on the functional images for any of the 33 participants.

3 The MNI coordinates of these activation peaks from previous studies are: Isik et al. (2017): 54, -43, 18; Landsiedel et al. (2022): 56, -46, 8 & -58, -50, 8; Walbrin et al. (2018): 52, -44, 18; Walbrin et al. (2020) adult group: 55, -44, 12 & -51, -51, 10.

4 These stick-light displays were the same as the point-light displays except that the dots were linked by full lines. The main findings reported by Sapey-Triomphe et al. (2017) and highlighted here were for the contrasts collapsed across the point-light and stick-light stimulus conditions.
